# Perception of Foreign and National Political Leaders in Russia

**DOI:** 10.3390/bs10060103

**Published:** 2020-06-19

**Authors:** Victor Petrenko, Olga Mitina, Marina Papovyan

**Affiliations:** Department of Psychology, Lomonosov Moscow State University, Mokhovaya st. 11/9, 125009 Moscow, Russia; victor-petrenko@mail.ru (V.P.); m.papovyan@gmail.com (M.P.)

**Keywords:** political perception, political leaders, image, social representation, psychosemantics

## Abstract

The present study aimed to determine the composition of factors that underlie the images of foreign and domestic political leaders among Russian youth and therefore play a major role in shaping social representations. The research was conducted within the theoretical and methodological framework of the psychosemantic approach, which seeks to reconstruct systems of meanings, both individual and collective, through the investigation of implicit as well as explicit categories of perception. The study comprised two stages, in which participants were administered a psychosemantic questionnaire to evaluate political leaders according to some professional and personal characteristics. The first part was conducted in 2015–2016 with a student sample (*n* = 147) using a set of political leaders from various countries. In the second part, carried out in 2017–2018, the participants (*n* = 200) also filled out a questionnaire, this time evaluating modern Russian leaders. A principal component analysis was performed on each of the data sets, revealing that two categories—namely, morality and professional characteristics—are present in both factor structures, whereas other factors are different. Several important theoretical and practical implications are discussed.

## 1. Introduction

Political leaders nowadays are often regarded as celebrities, with their personalities and private lives becoming the center of media and public attention. This increased interest in individual politicians is associated with the term “personalization”, which is used to describe a major trend in today’s politics, placing personality aspects above ideological positions and policy choices [1]. As a consequence, modern media and public discourse are largely centered around the personalities of political leaders.

Additionally, the issue of political perception seems to prove instrumental in bridging the theoretical and methodological divide in certain areas of political psychology research, such as the study of elite political behavior and the study of mass political behavior [2].

From this perspective, the study of the perception of political leaders emerges as one of the topical fields of psychological research. In recent decades, the study of political leaders in international relations has undergone substantial transformations, with political psychologists’ interest moving away from the study of operational codes or personality profiles [2]. Together with the changing views on leadership among academics (resulting, e.g., in defining the concept of charisma as a phenomenon of perception constructed by leaders and followers rather than a personality trait [3]), this has led to the notion of “image” gaining a predominant position in the study of political leaders. In the realm of international politics, for instance, the impact of the political leader’s image has been related to the “spillover effect” (an “image transfer between images of countries and images of sub-country entities”, in this case, political leaders, when these entities are ascribed similar traits) [4].

The crucial task set by political psychologists is to identify the key dimensions that are employed in political perception. Research in political communication has accumulated (typically through applying content analysis to the national election context [4]) a relatively voluminous, although rather loosely structured knowledge about dimensions used in the perception and evaluation of political leaders. Two dimensions mostly agreed upon are competence and integrity. Other dimensions, such as dynamism/strength/leadership, likeability, charisma, warmth, etc., have also been identified by researchers [5].

In their study of how a fictitious political leader’s image influences their home country’s image, S. Klein and D. Ingenhoff [4] organized the aforementioned image dimensions into the following system: the functional–cognitive image dimension (includes competence and leadership skills), the social–cognitive dimension (integrity), and the affective–expressive dimension (charisma). According to S. Pancer et al. [5], domestic leaders are also perceived according to the dimensions of charisma, competence, and integrity. However, the samples from three countries (the United Kingdom, Canada, and the United States) demonstrated systematic variety in the weights assigned to these dimensions in determining overall evaluations.

The study of a political leader’s image, therefore, is of utmost importance for political psychology. Analysis of criteria that determine the way political leaders are perceived and assessed is a necessary step to a better understanding of this psychological and political construct. There is no consensus on which categories are most significant, they are different in different countries and depend on socio-demographic, personality, and value characteristics [6,7].

Our objective was to identify the key dimensions according to which political leaders are perceived in a student sample. We operated within the conceptual framework of psychosemantics. Building on the insights into thought and speech processes gained by Soviet psychologists (L. Vygotsky, A. Leontiev, A. Luria), and the methodological frameworks of George Kelly’s and Charles Osgood’s research, psychosemantics seeks to reconstruct the systems of meaning, both individual and collective. Psychosemantic techniques provide access to implicit categories of consciousness, where reflection is rarely experienced. As conscious and unconscious processes play an equally influential role in political perception, application of psychosemantics enables researchers to analyze a wide variety of political phenomena and processes [8,9,10], and take into account not only explicit but also implicit levels of perception of a political leader, which is an important point for a comprehensive analysis [11].

In line with the commonly applied understanding of the political leader’s image as “a set of ideas and beliefs regarding the politically relevant characteristics” [4], we regard it as a psychological construct that exists in individual or mass consciousness within the psychosemantic space formed by certain dimensions of perception. Applying the notion of social representation as described by S. Moscovici [9] to our understanding of a political image, we can develop an appropriate methodology to study its formation and dynamics.

## 2. Materials and Methods

The study design had two parts. In each part, respondents were administered the psychosemantic questionnaire “Political Leader’s Image”, which has been successfully used in previous studies on political perception [10]. This technique makes it possible to explicate categorial structures that determine the perception of political leaders. The questionnaire taps into the implicit level of perception, thus providing a possibility to overcome the shortcomings of traditional self-report tools (e.g., social desirability bias). The aim is to produce a matrix whose columns represent political leaders and rows represent descriptor scales characterizing the political leaders according to a number of professional and personality traits. Each political leader is assessed on each characteristic with a Likert-type scale. The scores have a 7-point gradation (3, 2, 1, 0, −1, −2, −3), with number 3 being the maximum score for the suggested characteristic and number −3 representing the highest intensity (from the respondent’s point of view) of the opposite antonymous characteristic.

University students studying social and political science from the Kirov region of Russia took part in both parts of the study. Focusing on this particular sample, we were guided by the following considerations:

- Russia is a very big country with divergent opinions. As there is a huge political divergence among different regions of Russia, we decided to focus on a region which is “average” in social and political aspects. The level of material wellbeing and results in political elections are similar to average Russian statistics. Political preferences in the Kirov region are relatively homogenous and consistent with wider national preferences [12].
- University students are educated individuals with an arguably relatively high level of political sophistication. Their answers are more thoughtful and consequently less random and more likely to provide noise-free data. At the same time, they represent the views of people surrounding them. They are not an elite or narrow group with specific opinions.

In the first part, conducted in 2016, the participants (*n* = 147) evaluated 15 world leaders (see Appendix A for the list of names and short descriptions).

The list consisted of world political leaders, both contemporary and those regarded as symbolic figures, that are familiar to most Russian citizens. For instance, Hitler was included to set the value of “an absolute evil” in the semantic space. Additionally, respondents were asked to assess an “ideal” political leader. This image helps to identify the connotations attached to the items (characteristics) in the questionnaire, as well as to the categories (factors) that are produced by combining these items and form the semantic space.

The second study was carried out in late 2017 to early 2018. The participants (*n* = 200) were asked to assess 16 contemporary Russian political figures (see Appendix B for the list of names and short descriptions).

As was demonstrated in earlier psychosemantic studies, age and sex differences in the perception of political leaders are not very significant [13]. Although the samples cannot be considered representative, due to the sophisticated nature of the psychosemantic method it is possible to obtain meaningful results from skewed data.

The data sets were analyzed using SPSS (Version 15.0) (computer software, SPSS Inc.: Chicago, IL, USA) with principal component analysis (oblimin rotation with Kaiser normalization).

## 3. Results

### 3.1. Categorial Structures of Political Leaders’ Images

#### 3.1.1. Factors Underlying the Perception of World Leaders

The analysis has revealed four major factors (components) that explain 18.50, 9.75, 7.01, and 6.22% of the variance respectively. We provide the following interpretation of the factors (factor load for each item is put near this item):

The first factor—“***morality***”—includes such characteristics as:
His/her works contribute to higher morality in society0.78He/she works for the good of all humankind0.74Personally, I like him/her0.71He/she pursues peaceful policies0.70

In most studies, a similar factor is typically referred to as “integrity”—although the precise interpretation may differ (for example, in S. Klein and D. Ingenhoff’s study this dimension was measured by such items as “The prime minister is honest and keeps his/her promises”, “He/she sticks to his/her principles”, “He/she is very reliable” [4])—or “sanctity”, “fairness” [7].

A positive attitude towards Russia is also associated with morality. Service to the people is viewed as a significant component of moral conduct. Personal charm, wit, and physical attractiveness all contribute to this factor. It is worth noting that the set of characteristics that comprise the morality factor and are thus likely to be popular as policies include a focus on domestic resources, peace, and human rights. The “ideal leader” image gained a maximum score on this factor, which signifies the total approval of a leader’s moral conduct.

The second factor was defined as “***charisma***”. It included such items as:
He/she is a well-known politician0.68He/she makes bold decisions0.58He/she has a strong personality0.57He/she promotes the interests of the military–industrial complex0.55He/she has a magnetic personality0.55

According to studies in leadership, the emotional links between the leader and his/her followers have a strong impact on the political leader’s image. This is characteristic of the so-called charismatic leadership, based on emotions, in contrast to instrumental transactional leadership, which is characterized by a calculated notion of the leader’s efficiency. Charismatic leadership is prevalent in the majority of modern societies [3]. It might be worth noting that the perception of charisma may vary significantly depending on whether it was experienced first-hand [4].

From the point of view of the respondents, to be perceived as charismatic, a leader must be well-known. A lack of information about a political leader correlates with the perceived lack of charisma.

The third factor was interpreted as “***authoritarianism and dictatorship*** (with the orientation to socialist ideology)”, with characteristics including:
His/her political work leads to dictatorship0.67He/she is a proponent of communist (socialist) ideology0.56He/she is a proponent of a “firm hand policy”0.56He/she is a militarist0.55He/she is a proponent of a command economy0.52

Thus, for Russians, authoritarianism seems to be unrelated to morality. Additionally, along with authoritarianism and democracy as such, this construct includes nationalism and militarism, as well as socialist ideology, which for many respondents is associated with the totalitarian Soviet past. As the respondents clearly do not want an ideal political leader to possess this characteristic, it can be considered negative. These results are consistent with the results of other studies confirming that a positive attitude towards authoritarian leaders is more determined by individual traits and not by the cultural characteristics of the country [14].

The fourth factor—“***orientation toward globalism*** (with the orientation to the interests of inner elite circles)” included such characteristics as:
He/she promotes the interests of foreign capital0.59He/she advocates integration in world economy0.56He/she is a populist (promises a lot)0.52He/she believes that the development of the country should be based on external help0.51His/her political work leads to societal fragmentation, division of society into rich and poor0.50

Although the factor is unipolar, an implicit opposite pole can be interpreted as “isolationism”. Globalism implies following political and economic trends of the developed world powers, as well as promoting democracy and a market economy. The respondents might believe that such an orientation makes a political leader less autonomous, and its political implementation never does any good, as it is lucrative only for officials and widens the gap between the rich and the poor. This pole is formed by personal characteristics with negative connotations, demonstrating that the participants distrust this kind of person and therefore disapprove of this orientation: populism and grandstanding. For many Russians, liberal values have obtained negative connotations as a result of negatively viewed outcomes of the privatization of state-owned enterprises and the ensuing disproportionate economic inequality. The “ideal leader” image score on the fourth factor corresponds to mild isolationism, which indicates that the respondents are unwilling to live in a “global village”. This attitude is probably influenced by the official media positioning Russia in opposition to the West “with its desire to ensure its dominance and weaken Russia as the only state capable of challenging this hegemony”. It should also be noted that the variance of an ideal leader’s scores on the globalism factor is quite large, suggesting that the respondents’ opinions on this issue are not uniform.

#### 3.1.2. Factors Underlying the Perception of Russian Leaders

Four major factors (components) were obtained from the analysis, explaining 25.4, 6.0, 4.0 and 3.5% of the variance respectively. Factor loads are given in the brackets.

Similarly to the first study, the first factor was interpreted as “***morality***”. It includes such characteristics as:
He/she is able to sacrifice his/her own interests0.76His/her work improves the spirit of Russian society0.75His/her work contributes to scientific and cultural advances0.75He/she is honest0.73

The second factor—“***political adventurism***”—comprises two seemingly antonymous characteristics:
He/she is prone to snap decisions0.59He/she has a mind of his/her own0.58

One explanation for this rather strange mix could be derived from the body of studies focused on identifying the peculiarities of the political decision-making process—for example, the application of Kahneman and Tversky’s prospect theory to political context has demonstrated that the political decision-making process differs from commonly observed patterns [15]. Although the research in this area has not yet produced conclusive results, it is possible to suggest that on the implicit level the underlying explanatory frameworks of the specific nature of the political decision-making process can be captured by collective consciousness.

The third factor was interpreted as “***charisma***”, with such characteristics as:
He/she can influence the masses, grip the audience (a charismatic leader)0.54He/she has a strong personality0.53He/she makes bold decisions0.53He/she has a magnetic personality0.52

It should be noted that according to the results of our survey, a political leader must have a forceful, magnetic personality and stand their ground rather than submit to someone else’s authority. The notion of strength is one of the key parameters utilized for the assessment of political leaders in the political culture of post-Soviet Russia [16].

The fourth factor—“***statism***”—includes such characteristics as:
He/she promotes the interests of the military–industrial complex0.66He/she promotes the interests of the top bureaucracy0.56His/her work improves the country’s defense capabilities0.52In his/her political activity, he/she emphasizes the primacy of the state’s interests0.51

The ability to protect the interests of one’s country tends to be favorably regarded in the political culture of post-Soviet Russia as a representation of personal and political strength [16].

### 3.2. Positions of the Images of Political Leaders

**Figure 1:**
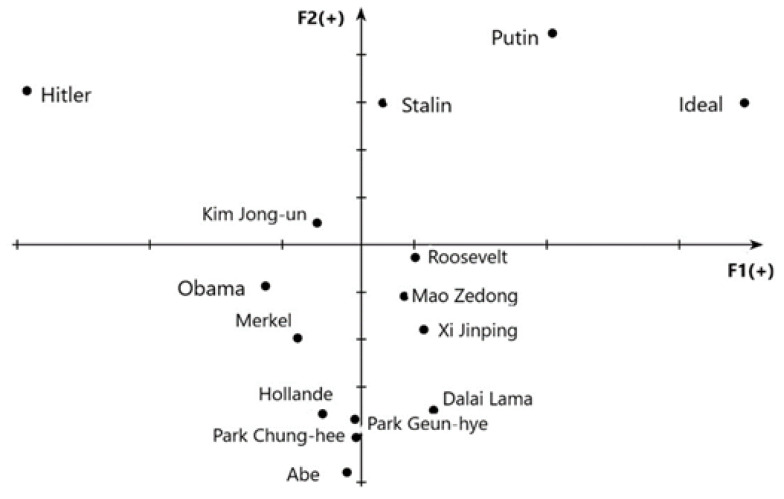
Positions of the images of world political leaders: F1—morality, F2—charisma.

**Figure 2:**
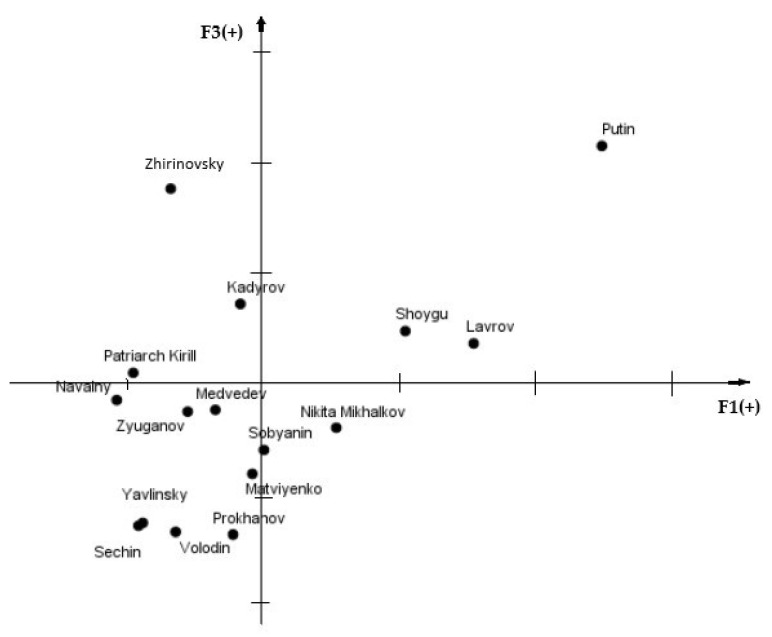
Positions of the images of Russian political leaders: F1—morality, F3—charisma.

To demonstrate how political leaders are perceived in comparison to one another, we identified the positions (i.e., coordinates) of political leaders in a multidimensional psychosemantic space. To provide a meaningful comparison, we chose two factors that were present in both categorial structures—“***morality***” (Factor 1 in both studies) and “***charisma***” (Factor 2 in Study 1, Factor 3 in Study 2). Figure 1 and Figure 2 demonstrate the resulting psychosemantic spaces.

#### 3.2.1. World Leaders

Figure 1 shows that Russians positively evaluate Vladimir Putin’s image on both charisma and moral criteria. In 2016 he was opposed by Obama, who received negative assessment on both criteria. Hitler demonstrates the most infamous image in terms of morality. Stalin’s image is perceived neutrally on morality and receives quite a high evaluation of charisma. (Hitler’s image also has a sufficiently high score on charisma.) The participants seem to have accepted to a certain degree the idea of these two political leaders ensuring “effective management” [17]. Political leaders that are least known in Russia are perceived by the respondents as least charismatic.

#### 3.2.2. Russian Leaders

Figure 2 also shows that Vladimir Putin is perceived considerably more positively on both criteria in comparison with other domestic political figures. Half of the politicians (8 out of 16) are viewed negatively on both moral and professional criteria.

## 4. Discussion

Thus, the results have demonstrated that the semantic spaces that describe the perception of the world and national political leaders include two similar factors—“***morality***” and “***charisma***”. These findings partially reproduce the data from previous research in the psychosemantics of political perception [8,10], as well as results from other authors [4,5]. However, while for international leaders the other two factors were interpreted as “***authoritarianism and dictatorship***” and “***globalism***”, Russian leaders were evaluated on “***political adventurism***” and “***statism***” categories. Overall, the results support the idea of the predominance of personality traits over political stance and party membership in the political leader’s image.

The categories of morality and charisma have positive connotations: they are ascribed to the image of an ideal political leader. The categories of authoritarianism, globalism, political adventurism, and statism have a weaker or negative connotation; their moderate presence is either tolerated (i.e., does not affect the integral image) or regarded as beneficial, but only to a certain degree.

According to the survey’s results in early 2018 (before Russia’s presidential election), Vladimir Putin’s image was perceived among university students as positive, close to ideal. Studies by other political psychologists, both Russian and foreign, confirm this fact [18,19].

In the majority of cases, evaluation of images matches the evaluation of these political leaders in the official media. For example, Obama’s negative image on the main characteristics, including morality, is formed by Russian media. At the same time, for many years these media outlets have been creating the image of Putin as an iron man with a big heart, capable of flying planes and riding horses, but also willing to respond to cries for help of those who have found themselves in a difficult situation.

It is worth noting that external attractiveness does not make a significant contribution to the assessment of the image and is not necessarily a component of charisma (compared with data obtained in other countries [20]). Finding out whether this is a specific feature of our sample or implicitly manifests itself in other qualities is the task of future research.

These days many political scientists, social analysts, and political journalists predict a rise in the perceived importance of professional qualities after the pandemic. This may result in actual professionals becoming leaders. There has been an increased demand for professional competence, as well as for scientific knowledge. However, the 2018 cross-section captured that charisma is perceived as being of higher significance. Moreover, it is people’s implicit perception that a charismatic leader is highly professional and competent. In Russia, the public is yet to see a professional become a public leader.

Additionally, we note that the shifting trends in the statism factor in summer 2018 should also have resulted in a decline in Putin’s approval rating.

Since morality and charisma are the main parameters in the perception and assessment of leaders in Russia, their decline may cause the approval ratings to dip. Many Russians see the raising of retirement age and disregard for people’s health for the sake of political interests as going against moral values. At the same time, members of the Russian opposition score political points by promoting the idea of justice and criticizing the authorities as amoral. It is thus possible, by studying the structure of Russian citizens’ political preferences, to predict who will succeed or fail.

This study, however, has some limitations. First of all, it is not based on a very big sample (two studies with a total of 347 respondents). However, psychosemantic research has its specificity. It enables researchers to identify deeper implicit perceptions and is less susceptible to social desirability bias, which is highly typical of political research in Russia. The flip side of this is that psychosemantic methods are closer to an in-depth interview, rather than a social survey, hence the sample volume limitations.

The second limitation is that the conclusions are limited to student samples, i.e., educated individuals with an arguably relatively high level of political sophistication. However, they are a suitable group in terms of relevance. These are future social sciences educators in schools, who will influence the political views of Russian citizens in the immediate future, so our results also possess a certain prognostic value. Furthermore, their answers are more thoughtful and consequently less random and more likely to provide noise-free data. Additionally, it was our conscious decision to choose a typical regional center, more representative of Russians’ political sentiments.

As there is a huge political divergence among different regions of Russia, between the two well-known capitals and the rest of Russia, which has even produced the notion of “two different Russias”, we intended to show the Russian “hinterland” in this study. A comparison of the perceptions of respondents in capitals and farther regions would be the aim of a separate study.

The third question is how representative is the set of items: how to ensure that the items of the questionnaire cover the entire phenomenology, and include all possible aspects of a leader’s image (to prove «substantive validity»). Certainly, there’s always a risk of failing to provide the full picture. We can always assume that something has been missed out. This is a methodological and philosophical problem. We create models, which are inherently incomprehensive and can always be expanded and improved. However, model designers always strive to maximize the explaining and predicting potential of a model based on their professional experience and expertise. We had been developing our list of questions for more than 20 years. The questions were selected through the analysis of the current political discourse in Russia and updated in each study so that they are relevant to the task of assessing political leaders’ images, reflect the actual range of opinions among respondents and represent the phenomenology of the perception of political leaders most adequately.

In conclusion, our findings have important theoretical and practical implications. Firstly, the results can improve our understanding of political processes by introducing an explanatory framework of political leaders’ perception. Secondly, from a practical standpoint, the obtained results may prove useful for public relations practitioners, particularly in the context of using political discourse as a vehicle for promoting power. With the aim of such common PR methods as framing being understood as “to select some aspects of a perceived reality and make them more salient in a communicating text to set a certain agenda” [21], the practical importance of taking into account the underlying structure of this perceived reality seems obvious. Understanding how the similarity between an individual’s perception of his/her personal characteristics and his/her perception of political leaders influences the development of political preferences can help to improve the level of civic participation and political culture among young people.

## Appendix A. List of World Political Leaders

*Vladimir Putin*	President of the Russian Federation (2000–2008, since 2012), The Prime Minister of the RF (1999–2000, 2008–2012).
*Barack Obama*	The 44th President of the United States (2009–2017).
*Park Geun-Hye*	The 18th President of South Korea (2013–2017).
*Kim Jong-un*	Supreme Leader of North Korea (since 2011).
*Xi Jinping*	President of the People’s Republic of China (since 2013).
*Park Chung-hee*	The 3rd President of South Korea (1963–1979).
*Adolf Hitler*	Führer of Germany (1934–1945), leader of the Nazi Party.
*Shinzō Abe*	The Prime Minister of Japan (2006–2007, since 2012).
*Joseph Stalin*	The General Secretary of the Communist Party of the Soviet Union (1922–1953).
The 14th *Dalai Lama* (Tenzin Gyatso)	The spiritual leader of Tibetan Buddhism.
*Mao Zedong*	The founding father of the People’s Republic of China, President of the Communist Party of China (1943–1976).
*Angela Merkel*	Chancellor of Germany (since 2005).
*François Hollande*	President of the French Republic (2012–2017).
*Franklin D. Roosevelt*	The 32nd president of the United States (1933–1945).

## Appendix B. List of Russian Political Leaders

*Vladimir Putin*	President of the Russian Federation (2000–2008, since 2012), The Prime Minister of the RF (1999–2000, 2008–2012).
*Dmitry Medvedev*	The Prime Minister of Russia (2012–2020), President of the Russian Federation (2008–2012).
*Valentina Matviyenko*	Chairwoman of the Federation Council (since 2011).
*Vyacheslav Volodin*	The 10th Chairman of the State Duma (since 2016).
*Vladimir Zhirinovsky*	Russian politician, leader of the Liberal Democratic Party of Russia (LDPR).
*Gennady Zyuganov*	Russian politician, leader of the Communist Party of the Russian Federation.
*Patriarch Kirill (Cyril)*	Russian Orthodox bishop.
*Grigory Yavlinsky*	Russian economist and politician, Chairman of the social-liberal Yabloko Party (1993–2008).
*Alexei Navalny*	Russian opposition leader.
*Nikita Mikhalkov*	A famous Russian filmmaker, actor, and head of the Russian Cinematographers’ Union, He is actively involved in politics and is known as a strong supporter of Russian President Vladimir Putin.
*Ramzan Kadyrov*	Head of the Chechen Republic (since 2011).
*Igor Sechin*	Deputy Prime Minister of Russia (2008–2012), CEO of Rosneft, the Russian state oil company (since 2012).
*Sergey Sobyanin*	Mayor of Moscow city (since 2010).
*Sergey Shoygu*	Minister of Defence of the Russian Federation (since 2012).
*Sergey Lavrov*	Minister of Foreign Affairs of the Russian Federation (since 2004).
*Alexander Prokhanov*	Russian writer and public figure, editor-in-chief of Russia’s ultra-right opposition newspaper “Zavtra”.
